# Supplementary figures and images for: Influence of social and physical environmental variation on antipredator behavior in mixed-species parid flocks
Source: PLoS One. 2023 Dec 21;18(12):e0295910. doi: 10.1371/journal.pone.0295910 (PMC10735029; doi:10.1371/journal.pone.0295910)

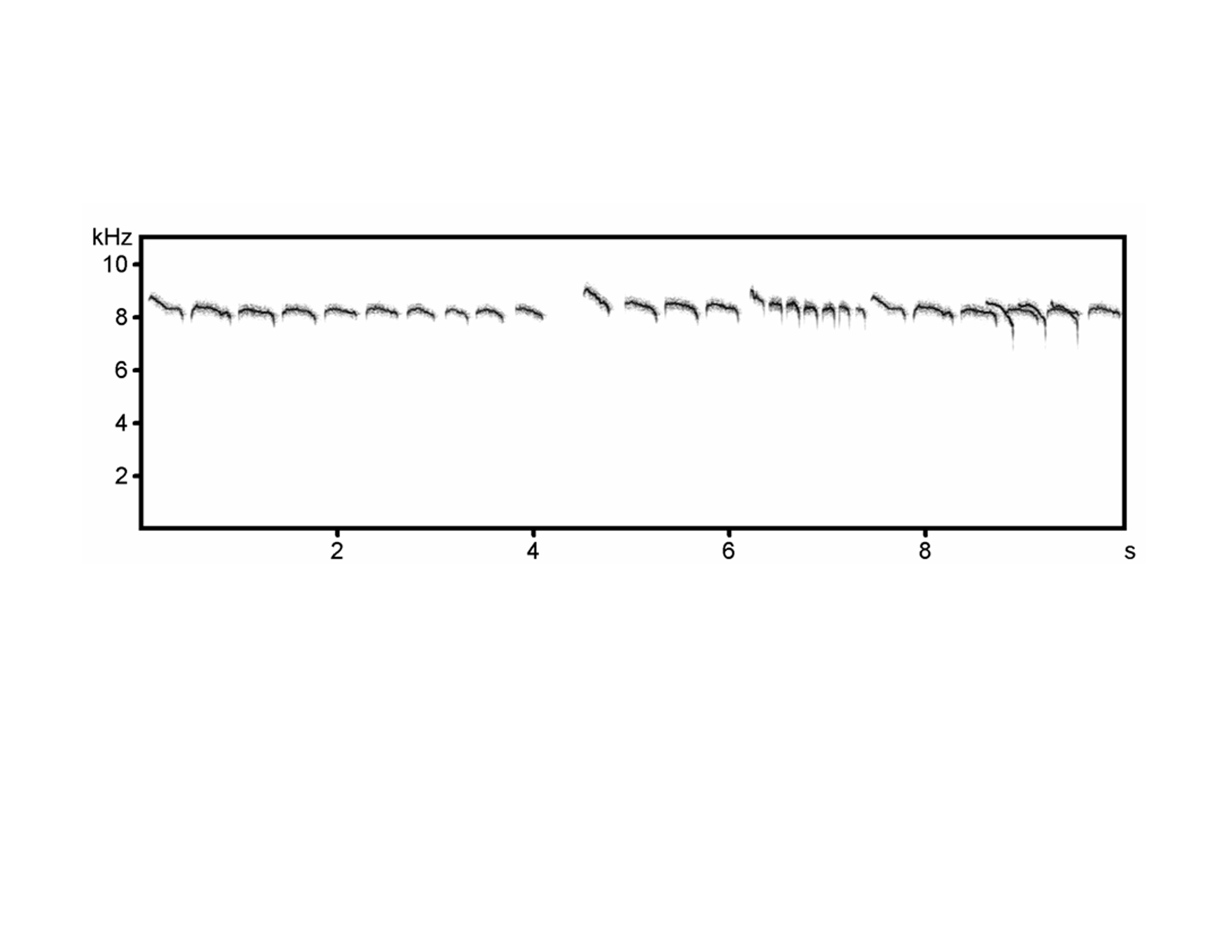

Supplement: S1 Fig — (TIF) [file pone.0295910.s001.tif]

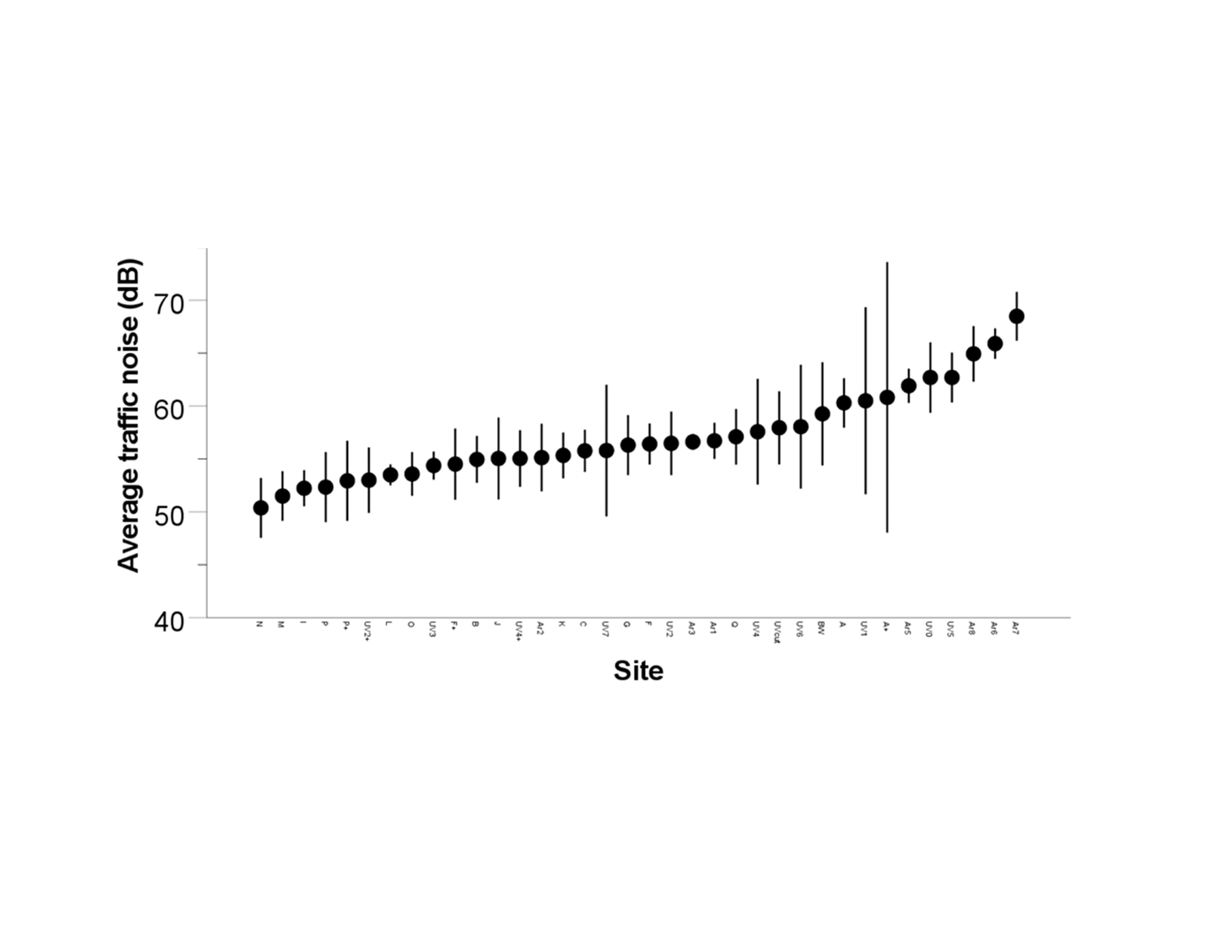

Supplement: S2 Fig — (TIF) [file pone.0295910.s002.tif]

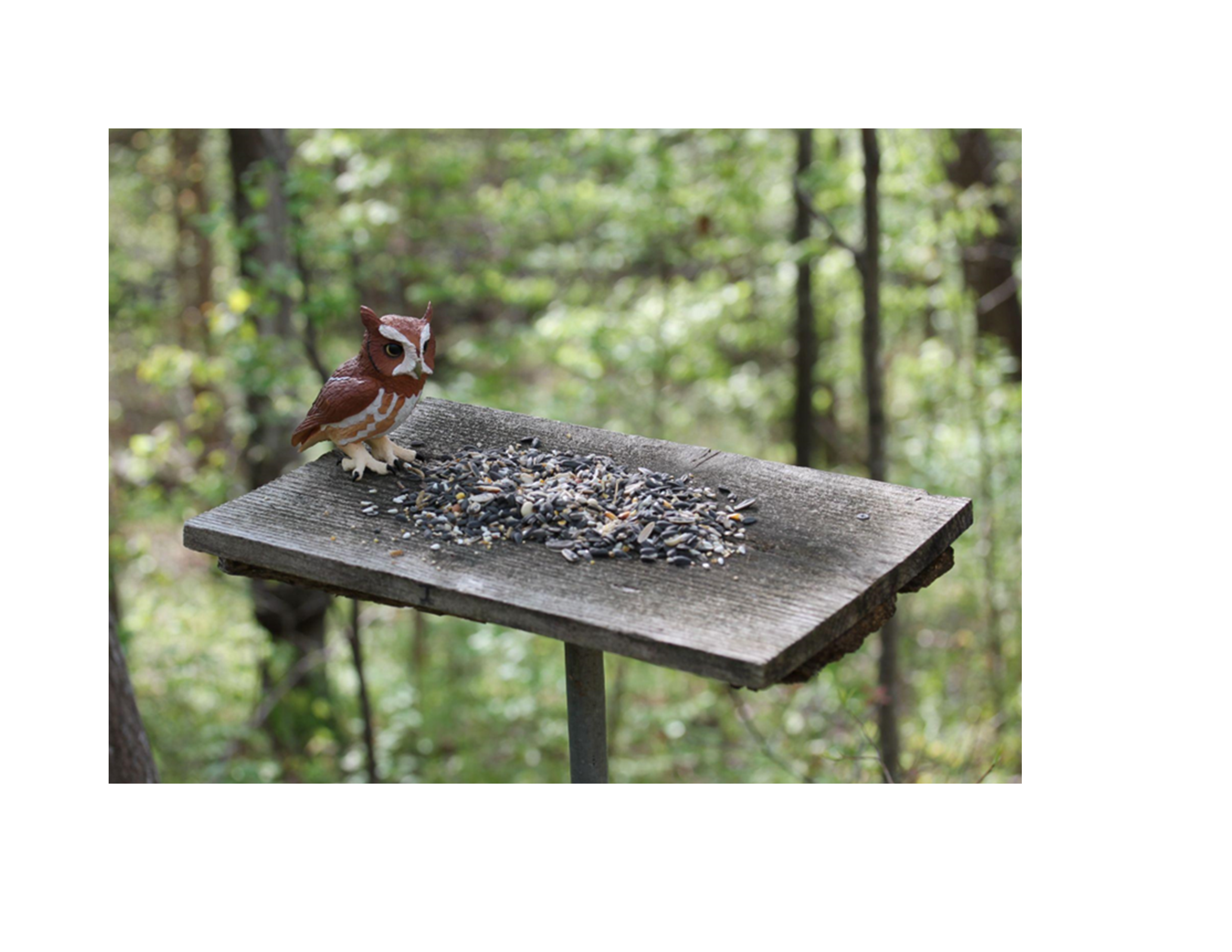

Supplement: S1 File — (TIF) [file pone.0295910.s003.tif]
